# The advanced strategy for enhancing biobutanol production and high-efficient product recovery with reduced wastewater generation

**DOI:** 10.1186/s13068-017-0836-7

**Published:** 2017-06-10

**Authors:** Chuang Xue, Xiaotong Zhang, Jufang Wang, Min Xiao, Lijie Chen, Fengwu Bai

**Affiliations:** 1 School of Life Science and Biotechnology, Dalian University of Technology, Dalian, 116024 China; 2 School of Bioscience & Bioengineering, South China University of Technology, Guangzhou, 510006 China

**Keywords:** Jerusalem artichoke stalk, Biobutanol, ABE fermentation, Vapor stripping–vapor permeation, Product recovery

## Abstract

**Background:**

Butanol as an important chemical and potential fuel could be produced via ABE fermentation from lignocellulosic biomass. The use of food-related feedstocks such as maize and sugar cane may not be a sustainable solution to world’s energy needs. Recently, Jerusalem artichoke tubers containing inulin have been used as feedstock for butanol production, but this bioprocess is not commercially feasible due to the great value of inulin as functional food. Till now, there is a gap on the utilization of Jerusalem artichoke stalk (JAS) as feedstock for microbial butanol production.

**Results:**

Biobutanol production from JAS was investigated in order to improve cellulose digestibility and efficient biobutanol fermentation. Compared with 9.0 g/L butanol (14.7 g/L ABE) production by 2% NaOH pretreatment of JAS, 11.8 g/L butanol (17.6 g/L ABE) was produced in the best scenario conditions of NaOH–H_2_O_2_ pretreatment, washing times and citrate buffer strengths etc. Furthermore, more than >64% water in washing pretreated JAS process could be saved, with improving butanol production by >25.0%. To mimic in situ product recovery for ABE fermentation, the vapor stripping–vapor permeation (VSVP) process steadily produced 323.4–348.7 g/L butanol (542.7–594.0 g/L ABE) in condensate, which showed more potentials than pervaporation for butanol recovery.

**Conclusions:**

Therefore, the present study demonstrated an effective strategy on efficient biobutanol production using lignocellulosic biomass. The process optimization could contribute to significant reduction of wastewater emission and the improvement of lignocellulosic biomass digestibility and biobutanol production, which makes biobutanol production more efficient using JAS.

## Background

With the gradual exhaustion of fossil fuels like coal, petroleum, and natural gas in the earth, more attentions have been paid on biofuels production derived from renewable biomass [1]. Butanol as an important chemical and potential fuel could be produced via ABE fermentation using maize, sugar cane, etc., but the use of these food-related feedstocks to produce butanol may not be a sustainable solution to world’s energy needs [2, 3]. Biofuels, such as bioethanol and biobutanol, can be produced in large scale from lignocellulosic biomass due to its massive amount in the world [4].

Jerusalem artichoke (JA) is a perennial crop of the Composite family, which can be planted in marginal lands without competing for arable land with grain crops [5, 6]. It is a dedicated energy crop, which can tolerate various environmental stresses such as drought, salt, pest invasion, and infection of plant diseases. Jerusalem artichoke stalk (JAS), like other lignocellulosic resources, consists of a rigid cellulose structure combined with amorphous hemicellulose and a lignin cross-linked structure [7, 8], which makes the JAS pretreatment exceedingly difficult for biofuels production. Recently, Jerusalem artichoke tubers containing inulin have been used as feedstock for butanol production, but this bioprocess is not commercially feasible due to the great value of inulin as functional food [9]. Till now, there is a gap on the utilization of JAS as feedstock for microbial butanol production.

Lignocellulosic biomass is required to be pretreated and enzymatically hydrolyzed into fermentable sugars for butanol fermentation. Nowadays, acid or alkali pretreatment has been extensively studied for cellulosic butanol production [10, 11]. Addition of an oxidant agent (oxygen/H_2_O_2_) into alkaline pretreatment (NaOH/Ca(OH)_2_) of wheat straw can improve the performance by favoring lignin removal [12, 13], but NaOH–H_2_O_2_ pretreatment of JAS has been never tested before. Therefore, the JAS pretreatment method for biocompatibility with butanol fermentation needs further exploration. In addition, alkaline and oxidative pretreatments tend to generate aromatic compounds (i.e., phenolics) as well as acetyl derivatives, which are considered as inhibitory compounds of microbial fermentation [14]. Following NaOH–H_2_O_2_ treatment, the water washing for pH adjustment could remove above-mentioned inhibitory compounds [15], but this process will inevitably generate a large amount of industrial wastewater and increase environmental burden.

Even though lots of efforts on strain development have been made by genetically engineering *Clostridium* spp. and heterogeneous strains, butanol concentration in fermentation broth could not exceed 2% (w/v) due to the limited stress tolerance of strains [2, 3, 16–19]. Since conventional distillation is energy intensive for butanol purification, several alternative techniques such as gas stripping, liquid–liquid extraction, pervaporation (PV), and adsorption have received increasing attention as they could continuously remove ABE solvents from fermentation broth and reduce the inhibition of ABE to cells by integrating with ABE fermentation [20–22]. The vapor stripping–vapor permeation (VSVP) process, termed membrane-assisted vapor stripping, was more rarely studied than pervaporation and gas stripping for butanol recovery, which could prevent membrane fouling due to volatilized organic compounds contacting both sides of the membrane during mass transfer [23, 24]. Furthermore, the VSVP process has superior butanol selectivity as it combines the advantageous merits of pervaporation and gas stripping [23].

In this study, JAS was firstly used for biobutanol production, with investigating different concentrations of NaOH–H_2_O_2_ pretreatments for improving lignin removal rate and fermentable sugar release. Furthermore, the conditions such as treatment time during alkaline pretreatment, washing times after pretreatment, citrate buffer in enzymatic hydrolysis, and initial pH for butanol fermentation were also investigated for regulating the biocompatibility of JAS hydrolysate with butanol fermentation. To be highlighted, water washing times were firstly studied with aiming to reduce wastewater generation as well as improve butanol production. Finally, the VSVP and PV processes were compared to recover butanol from fermentation broth, and the VSVP process showed more potential in biobutanol production from JAS.

## Results and discussion

### Pretreatment of JAS with NaOH–H_2_O_2_

Different proportions of NaOH or/and H_2_O_2_ were used for JAS pretreatment, and the contents of cellulose, hemicelluloses, lignin are evaluated and summarized in Table 1. The composition of raw JAS used in this work contained 47.1% cellulose, 16.2% hemicellulose, and 24.2% lignin. Other constituents such as inulin, ash, and extractable constituents were ~12.5%. Compared with 20.1% of weight loss in 2% NaOH pretreatment, more weight losses of 24.8–28.3% occurred in 2% NaOH pretreatment combined with 3–9% H_2_O_2_. With the increase of H_2_O_2_ pretreatment from 0 to 9% (v/v), hemicellulose and lignin in the pretreated JAS decreased from 14 and 10.0% to 11.9 and 5.2%, respectively, indicating that the H_2_O_2_ in NaOH solution significantly facilitated hemicellulose and lignin release from solid JAS and solubilization in the alkaline solution. Correspondingly, when H_2_O_2_ supplementation was in the range from 0 to 6%, cellulose content in pretreated solid JAS increased from 55.1 to 64.0%, and there was no increase of cellulose with more H_2_O_2_ supplementation (9%). It was indicated that 2% NaOH–6% H_2_O_2_ treatment could allow more hemicellulose and lignin to dissolve in the alkaline solution, facilitating more cellulose saccharification in the subsequent enzymatic hydrolysis due to the removal of more lignin.

**Table 1:** Compositions of the untreated and pretreated JAS

Pretreatment	Weight loss (%)	Cellulose (%)	Cellulose removed (%)	Hemicellulose (%)	Hemicellulose removed (%)	Lignin (%)	Lignin removed (%)
Raw JAS	–	47.1 ± 0.1	–	16.2 ± 0.2	–	24.2 ± 0.6	–
2%NaOH	20.1 ± 3.1	55.1 ± 0.1	6.5 ± 0.4	14.0 ± 0.6	31 ± 0.5	10.0 ± 0.7	66.9 ± 0.7
4%NaOH–3% H_2_O_2_	26.9 ± 2.2	61.5 ± 1.4	4.6 ± 0.9	13.2 ± 0.7	40.4 ± 0.4	7.3 ± 0.4	77.9 ± 0.3
2%NaOH–3% H_2_O_2_	24.8 ± 1.7	59.3 ± 0.8	5.3 ± 0.7	13.5 ± 0.6	37.3 ± 0.7	9.1 ± 0.4	71.7 ± 0.5
2%NaOH–6% H_2_O_2_	27.3 ± 2.0	64.0 ± 0.2	1.2 ± 1.1	11.7 ± 0.2	47.5 ± 0.5	5.3 ± 0.6	84.1 ± 0.5
2%NaOH–9% H_2_O_2_	28.3 ± 1.5	64.3 ± 0.1	2.1 ± 0.7	11.9 ± 0.8	47.3 ± 0.2	5.2 ± 0.3	84.6 ± 0.4

The fermentability of pretreated biomasses for butanol production depends on fermentable sugars by enzymatic hydrolysis. To determine the amount of fermentable sugars from the pretreated JAS, the enzymatic hydrolysis using 20 FPU/g cellulase for 72 h was performed using the H_2_O_2_–NaOH pretreated JAS. As shown in Table 2, when H_2_O_2_ concentration in NaOH solution increased from 0 to 6% (v/v), glucose released from the pretreated JAS increased from 36.5 to 48.3 g/L, respectively. Then glucose decreased to 45.4 g/L with 9% H_2_O_2_ addition. Therefore, in the test range of H_2_O_2_ addition, 6% H_2_O_2_ addition in 2% NaOH solution could be able to produce the highest amount of the fermentable sugars (48.3 ± 0.5 g/L glucose; 11.6 ± 0.2 g/L xylose). Under alkaline conditions, hydrogen peroxide is dissociated to generate the hydroperoxyl anion (HOO−), which degrades lignin by reacting with the quinone structures of lignin, and carbonyl group of side chains or the double bonds in lignin [13].

**Table 2:** The performance of enzymatic hydrolysis and ABE fermentations using JAS hydrolysate pretreated by different NaOH–H_2_O_2_ concentrations

	2%NaOH	4%NaOH–H_2_O_2_ (3%, v/v)	2%NaOH–H_2_O_2_ (3%, v/v)	2%NaOH–H_2_O_2_ (6%, v/v)	2%NaOH–H_2_O_2_ (9%, v/v)
Initial glucose, g/L	36.5	41.6	40.3	48.3	45.4
Initial xylose, g/L	9.2	11.0	10.5	11.6	11.1
Initial Cellobiose, g/L	3.9	3.5	5.0	4.2	5.6
Initial Arabinose, g/L	2.4	2.3	2.5	2.0	2.1
Residual glucose, g/L	3.3	3.5	6.0	11.1	7.3
Residual xylose, g/L	2.0	1.2	2.6	2.7	2.5
Maximum OD	2.1	2.5	2.4	2.6	2.2
Fermentation time, h	60	60	60	60	60
Acetone, g/L	5.8	5.3	4.9	6.0	5.2
Ethanol, g/L	0.14	0.14	0.13	0.16	0.15
Butanol, g/L	9.0	10.2	9.7	11.0	10.5
Total ABE, g/L	14.7	15.5	14.4	17.0	16.3
Butanol yield, g/g	0.22	0.22	0.23	0.24	0.23

In order to study the fermentability of NaOH–H_2_O_2_ pretreated JAS hydrolysate, batch butanol fermentation was performed with *Clostridium beijerinckii* CC101. When the concentration of H_2_O_2_ ranged from 0 to 6%, butanol and ABE concentrations increased from 9.0 and 14.7 g/L to 11.0 and 17.0 g/L, respectively, but then gradually decreased to 10.5 and 16.3 g/L when the concentration of H_2_O_2_ increased to 9%. The maximum butanol concentration and yield of 11.0 g/L and 0.24 g/g were obtained when 2%NaOH–6%H_2_O_2_ solution was used for the pretreated JAS. The 2%NaOH–6%H_2_O_2_ pretreatment was not only beneficial for more fermentable sugars release, but also contributed to the best performance of butanol production. Thus, 2%NaOH–6%H_2_O_2_ pretreatment as the optimal alkaline condition was used in the following studies.

### Effect of NaOH–H_2_O_2_ pretreatment time on enzymatic hydrolysis

**Fig. 1:**
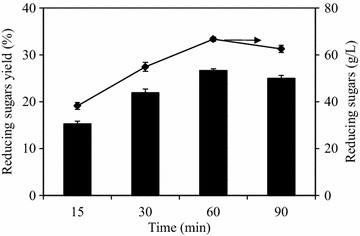
The concentrations and yields of reducing sugars at different treatment time by NaOH–H_2_O_2_ pretreatment

Since more hemicellulosic and cellulosic sugars during enzymatic hydrolysis preferred to be preserved, lower temperature at 121 °C was selected for NaOH–H_2_O_2_ pretreatment [25]. The concentrations of reducing sugars at different treatment time are investigated in Fig. 1. Reducing sugars increased from 38.2 to 66.7 g/L, with increasing the treatment time from 15 to 60 min, and then decreased to 62.6 g/L at 90 min. When treatment time was at 60 min, the highest yield and the concentration of reducing sugars were 26.7% and 66.7 g/L, increasing by 80.0 and 74.6%, respectively, compared with those at 15 min. Enough pretreatment time is required for allowing more small cellulose fibers exposed on the pretreated surface of JAS, which may improve the hydrolysis of cellulose [26, 27]. On the other hand, the increase in treatment time leads to lignin degradation in the severe conditions [28]. Excessive treatment time may lead to more inhibitory products generation. Therefore, the pretreatment time is essential for improving reducing sugar conversion efficiency of JAS.

### Effect of washing times on ABE fermentation and wastewater generation

Alkaline/hydrogen peroxide pretreatment of lignocellulosic materials could lead to high sugar yield with moderate temperature and pressure [29, 30]. However, after NaOH–H_2_O_2_ pretreatment, the pretreated biomass needs to be washed with water for removal of residual NaOH and inhibitory products. Moreover, lignin-derived phenolic compounds in the viscous alkali waste may inhibit cellulase hydrolysis and further butanol fermentation [31]. Abundant water is required for washing and removing these compounds, until the wastewater was neutral. Till now, it is not clear that how much water is required in consideration of both water saving and butanol production.

**Fig. 2:**
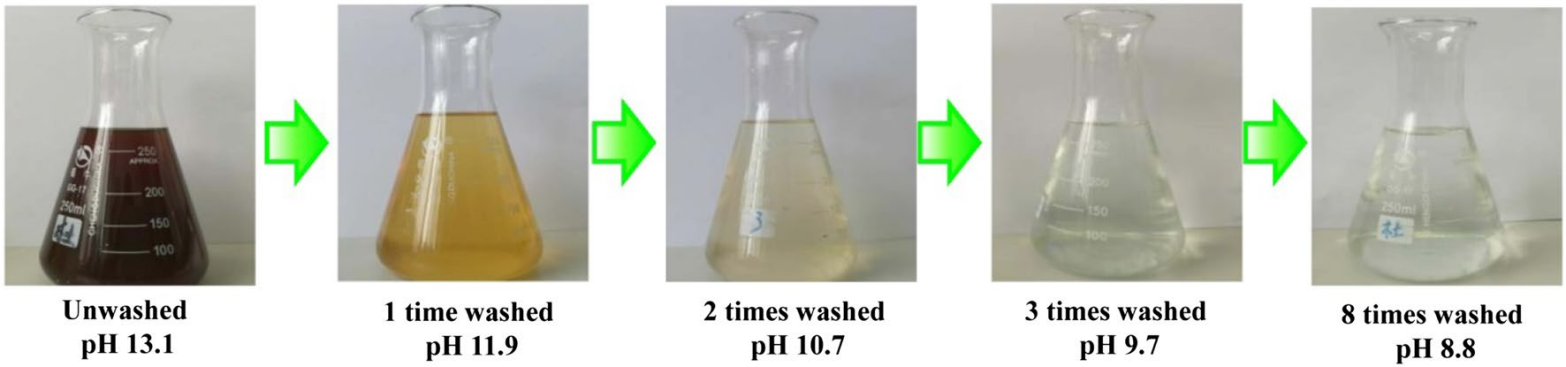
The pH and color variation of wastewater from washing for NaOH–H_2_O_2_ pretreated JAS

The effects of washing times on ABE fermentation and wastewater generation are shown in Table 3 and Fig. 2. The JAS was pretreated with NaOH–H_2_O_2_ and then washed from 0 to 8 times, respectively. With the increase of washing times from 0 to 8, the pH decreased from 13.1 to 8.8, respectively. As shown in Fig. 2, the color of wastewater from washing the NaOH–H_2_O_2_ pretreated JAS became gradually lighter with increasing the washing times. When the washing times were more than 3, the wastewater was almost colorless. It was difficult to lower the pH to neutral despite more water utilization. In most of previous studies, dozens of washing times were required for removing residual NaOH, the process of which generated >10 times volumes of wastewater than fermentation broth [32]. Therefore, in present study, the combined water washing/HCl-neutralization (HN) strategy was investigated with aim to reduce water utilization. As shown in Table 3, more washing times were beneficial for improving final butanol concentration in fermentation broth. When washing times increased from 0 to 8 times, butanol concentration in fermentation broth increased from 8.6 to 11.2 g/L, respectively, indicating that the increased washing times could remove more inhibitory products and make hydrolysate more biocompatible with the strain. Reducing sugars (glucose and xylose) also increased from 45.3 to 61.8 g/L, indicating that more washing contributed to additional sugars released from JAS during enzymatic hydrolysis. However, the highest butanol and ABE yields of 35.5 mg/g-JAS and 54.2 mg/g-JAS were achieved with 3 times washing and HCl neutralization. Butanol and ABE yields decreased slightly with 8 times washing due to gradually JAS weight lost after every time water washing. In our previous study, it was found that a large amount of water was required for washing the pretreated corn stover to neutral pH, which finally increased wastewater emission and production cost [23]. In present study, compared to 3.6 L water consumption/*g*-butanol in a conventional water-wash process (8-W), washing 3 times (3-W)/HN process significantly reduced water consumption by >64%. In general, the tradeoff between water consumption and butanol concentration is valuable for microbial butanol production from lignocellulosic biomass. The improved butanol concentration significantly contributes to the reduced product recovery cost by conventional distillation [20, 33]. In consideration of butanol concentration, more water is preferable for providing a suitable environment for fermentation strain by completely removing inhibitory products and residual NaOH. In present study, it was clear that the highest butanol and ABE yield were achieved with (3-W)/HN process, which resulted in the reduced wastewater emission and raw JAS cost. Consequently, the washing to neutral pH process may not be strictly necessary when applying the demonstrating NaOH–H_2_O_2_ pretreatment. The comparison of various pretreatment methods for JAS is shown in Table 4. In general, alkali pretreatment method could give higher sugars yield compared to acid pretreatment [34–37]. But a large amount of water is required for NaOH removal and neutralization. The demonstrating strategy could save washing water and give a high sugars yield. In summary, for environmental protection, the limited water washing combined with insignificant amount of HCl neutralization could be an effective bioprocess strategy for reducing the cost of butanol production.

**Table 3:** The effects of washing times on ABE fermentation and wastewater generation

	(0-W)/HN	(1-W)/HN	(2-W)/HN	(3-W)/HN	8-W
Initial pH	13.1	13.1	13.1	13.1	13.1
Washed/HCl-neutralized pH	13.1/8.8	11.9/8.8	10.7/8.8	9.7/8.8	8.8/8.8
Initial JAS, g	200	200	200	200	200
Pretreated JAS, g	172.7	170.1	165.6	162.3	148.6
Initial glucose, g/L	33.0	36.5	38.8	46.3	48.2
Initial xylose, g/L	12.3	12.5	12.4	13.9	13.6
Butanol, g/L	8.6	9.7	10.1	10.8	11.2
Acetone, g/L	4.8	4.9	5.2	5.7	5.8
Ethanol, g/L	0.2	0.2	0.2	0.2	0.2
Butanol yield, mg/g-JAS	29.7	33.0	33.5	35.5	33.5
ABE yield, mg/g-JAS	47.0	50.3	51.3	54.2	51.1
Water volume, L	0	3	6	9	24
Water consumption, L/g-butanol	0	0.45	0.90	1.3	3.6

*(0-W)/HN* unwashed/HCl-neutralization, *(1-W)/HN* washing 1 time/HCl-neutralization, *(2-W)/HN* washing 2 times/HCl-neutralization, *(3-W)/HN* washing 3 times/HCl-neutralization, *8-W* washing 8 times

**Table 4:** The comparison of various pretreatment methods for JAS

Pretreatment	Parameters	Water washing^a^ (g/g-JAS)	Sugars yield (g/g-JAS)	Comments	Refs.
Alkali	2% (w/v) NaOH, 121 °C, 1 h, washing to neutral pH	>240	0.23	A large amount of water for neutralization	[29]
Acid/alkali	0.5 % (v/v) H_2_SO_4_, 121 °C, 1 h/4% (w/v) NaOH, 121 °C, 1 h, washing to neutral pH	>260	0.33	A large amount of water for neutralization, high sugars yield, high energy cost	[30]
Acid	1% (v/v) H_2_SO_4_, 130 °C, 1.5 h	–	–	Low sugars yield, water saving	[31]
Acid	0.5% (v/v) H_2_SO_4_, 121 °C, 1 h	–	0.18	Low sugars yield, water saving	[32]
Alkali	4% (w/v) NaOH, 121 °C, 1 h washing to neutral pH	>260	0.26	A large amount of water for neutralization, high sugars yield	[32]
Alkali/peroxide	2% (w/v) NaOH–6% (v/v) H_2_O_2_, 121 °C, 1 h, washing to pH 8.8	45	0.27	Water saving, high sugars yield	This work

^a^Water utilization was calculated based on NaOH removal and neutralization

### Effect of citrate buffer on enzymatic hydrolysis and ABE fermentation

The commonly used citrate buffer strength for optimal cellulase activity is 50 mM [38, 39]. It is not clear that whether this designated strength for enzymatic hydrolysis is also optimal for subsequent butanol fermentation. In order to optimize buffer strength amenable to ABE fermentation, the effect of citrate buffer concentration on enzymatic hydrolysis and ABE fermentation using JAS was investigated (Table 5). When 20 g of JAS pretreated from NaOH to H_2_O_2_ was added to 100 mL of sodium citrate buffer in a concentration range of 20–100 mM, reducing sugars increased from 14.3 to 64.5 g/L, indicating that the citrate buffer strength has significant effect on fermentable sugars released from JAS.

**Table 5:** The performance of ABE fermentation with various citrate buffer strengths using JAS

	Sodium citrate buffer strengths (mM)				
	20	40	60	80	100
Initial glucose, g/L	8.0	21.9	45.9	47.2	48.9
Initial xylose, g/L	6.3	9.6	14.3	14.6	15.6
Reducing sugars, g/L	14.3	31.5	60.2	61.8	64.5
Residual glucose, g/L	0.1	0.4	11.7	10.7	10.9
Residual xylose, g/L	0.1	0.3	1.7	2.0	3.6
Maximum OD	1.7	1.8	2.2	1.8	1.8
Fermentation time, h	60	60	60	60	60
Acetone, g/L	1.0	3.0	5.4	5.2	5.0
Ethanol, g/L	0.15	0.10	0.17	0.18	0.17
Butanol, g/L	4.0	7.4	11.2	10.1	9.4
Total ABE, g/L	5.2	10.6	16.8	15.4	14.6
Butanol yield, g/g	0.28	0.24	0.24	0.21	0.19
Total ABE yield, g/g	0.37	0.34	0.36	0.31	0.29

When sodium citrate concentrations in the hydrolysate increased from 20 to 60 mM, butanol and ABE concentration increased from 4.0 and 5.2 g/L to 11.2 and 16.8 g/L, respectively, but then gradually decreased to 9.4 and 14.6 g/L when sodium citrate in the hydrolysate increased to 100 mM. The maximum concentrations of butanol and ABE were 11.2 and 16.8 g/L, respectively, when 60 mM citrate buffer was used for enzymatic hydrolysis. Under the scenario with 60 mM citrate buffer, more reducing sugars were consumed in ABE fermentation. The maximum cell growth was obtained in the JAS hydrolysate medium with 60 mM citrate buffer. Higher citrate strengths may inhibit cell growth by reducing the cells internal pH and proton motive force, and changing cell membrane permeability [40]. In addition, higher concentration of undissociated citric acid and higher medium osmolality also directly affect cell growth [23]. The demonstrating results above indicated that the enzymatic hydrolysis with 60 mM citrate buffer was more suitable for microbial butanol production using JAS hydrolysate. In addition, the sugars yield using Youtell’s cellulase in present study is 0.27 g/g-JAS, leading to the overall butanol yield of ~5% (w/w) from JAS. The sugars and butanol yields could be significantly improved when using Novozymes’s cellulase for enzymatic hydrolysis of JAS, which makes ABE production more competitive.

### The initial pH of the hydrolysate for ABE fermentation

In ABE fermentation, the initial pH of the hydrolysate has been recognized to be extremely important for butanol production. Due to the composition differences between JAS and other feedstocks such as corn stover, the optimal pH of the JAS hydrolysate is still not investigated for butanol production. As shown in Table 6, the initial pH in the range of 5.8–7.0 was evaluated for JAS hydrolysate. Reducing sugar of 58 ± 1.7 g/L for ABE fermentation was achieved with 20% NaOH–H_2_O_2_ pretreated biomass loading. The maximum cell growth was obtained with the initial pH of 6.0–6.2. When the pH was at 6.2–6.4, butanol concentrations were more than 11.0 g/L. The maximum butanol and total ABE were 11.8 and 17.6 g/L at the optimal pH of 6.2. The butanol yield and productivity were 0.25 g/g and 0.14 g/L/h, respectively, which were higher than those of other pH conditions. The performance of ABE fermentation was not satisfactory when the pH was below or above 6.2. During ABE fermentation, the rapid formation of the organic acids (acetic acid and butyric acid) resulted in a decrease of the pH in the hydrolysate. Solventogenesis starts when the pH reaches a “break point,” after which acids are re-assimilated and butanol and acetone are produced [41]. The optimal initial pH may be different due to the selection of raw materials, pretreatment methods, and strains. For example, it was reported that the optimal pH for liquefied corn stalks was 6.7 for achieving maximum yields of butanol and ABE [41]. Therefore, it is worthwhile to determine the optimum pH as well as other conditions for biobutanol production derived from the JAS feedstock.

**Table 6:** The performance of ABE fermentation with different initial pH of hydrolysate

	Initial pH of the hydrolysate						
	5.8	6.0	6.2	6.4	6.6	6.8	7.0
Initial reducing sugars, g/L	58.9	59.8	57.5	59	58.7	58	59.7
Residual sugars, g/L	12.6	10.3	10.4	10.0	9.5	13.2	14.4
Maximum OD	2.1	2.5	2.5	2.2	2.2	2.2	2.1
Fermentation time, h	60	60	60	60	60	60	60
Acetone, g/L	5.3	5.6	5.7	5.6	5.4	5.4	5.2
Ethanol, g/L	0.1	0.2	0.2	0.2	0.2	0.2	0.1
Butanol, g/L	9.7	10.0	11.8	11.1	10.8	10.5	10.0
Total ABE, g/L	15.1	15.8	17.6	16.9	16.3	16.0	15.3
Butanol yield, g/g	0.21	0.20	0.25	0.23	0.22	0.23	0.22
Butanol productivity, g/L/h	0.12	0.12	0.14	0.13	0.13	0.13	0.12

### Simulation of in situ product recovery during ABE fermentation

**Fig. 3:**
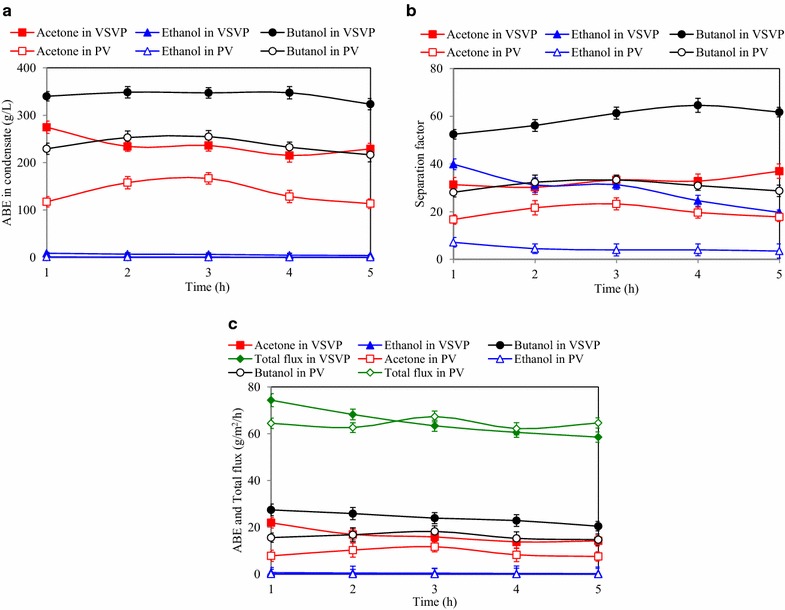
The performance of in situ ABE recovery using VSVP and PV processes. **a** ABE concentrations in condensate; **b** ABE separation factors; **c** ABE and total fluxes

To mimic in situ product recovery during ABE fermentation, the VSVP and PV processes were conducted using fermentation broth from ABE fermentation using JAS hydrolysate, respectively. The PV process is a membrane technique for liquid/liquid separation that has been extensively studied in recent years [20, 42]. The VSVP process is more advanced membrane-based technology than PV in which the solvent mixture vaporizes by gas stripping and then separates by the membrane [23]. The fermentation broth in 500 mL contained 11.8 g/L butanol, 5.7 g/L acetone, and 0.2 g/L ethanol, which was produced in best scenario above. Since the volume of feed fermentation broth was much higher than the recovered volume per hour, the performance of the VSVP process was very stable due to the ABE concentrations in feed solution maintaining at stable level. As can be seen in Fig. 3, when VSVP process was carried out in 5 h at 37 °C, the condensate containing 323.4–348.7 g/L butanol, 215.1–236.4 g/L acetone and 4.2–8.9 g/L ethanol was produced, with average butanol, acetone, and ethanol separation factors of 58.6, 33.6, and 29.8, respectively. The butanol flux and total flux were 22.6–25.5 and 58.6–74.3 g/m^2^/h, respectively.

In comparison, the PV process with the same fermentation broth at 37 °C produced the condensate containing 216.6–255.0 g/L butanol, 113.5–117.6 g/L acetone, and 0.6–1.2 g/L ethanol (Fig. 3). The average separation factors of butanol, acetone, and ethanol were 30.7, 20.1, and 5.4, respectively. The butanol flux and total flux were relatively stable in the range of 14.7–18.3 and 62.2–67.4 g/m^2^/h, respectively. In comparison with the PV process, the butanol separation factor of VSVP process was about twofold higher than that of PV process. Therefore, more concentrated butanol (ABE) could be achieved using VSVP process integrated with ABE fermentation.

### Complete product recovery from fermentation broth in batch mode

**Fig. 4:**
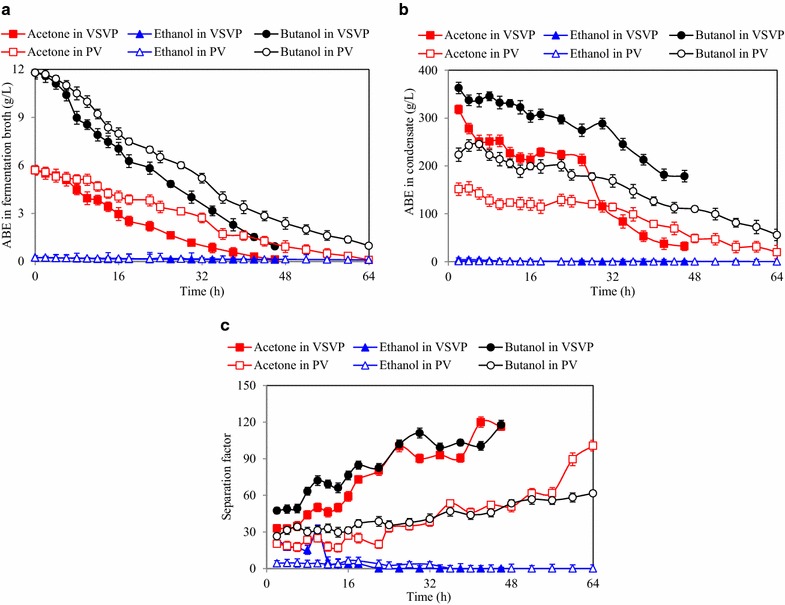
The performance of ABE recovery using VSVP and PV processes in batch mode. **a** ABE concentrations in fermentation broth; **b** ABE concentrations in condensate; **c** ABE separation factors

In order to completely recovery butanol/ABE from fermentation broth, product recovery from fermentation broth containing 11.8 g/L butanol, 5.7 g/L acetone, and 0.2 g/L ethanol in 500 mL was conducted in batch mode to evaluate the performance of VSVP and PV process, respectively. For VSVP process, within 46 h, butanol, acetone, and ethanol in fermentation broth decreased from 11.8, 5.7, and 0.2 to 0.9, 0.1, and 0.1 g/L, respectively (Fig. 4). With decreasing the butanol, acetone, and ethanol concentration in fermentation broth, the butanol, acetone and ethanol concentration in condensate decreased from 332.3 g/L, 278.3 g/L, and 4.2 g/L to 178.5 g/L, 33.0 g/L, and 0.6 g/L, respectively. However, the butanol and acetone separation factors gradually increased to the maximum levels of 117.7 and 116.3, respectively. The recovery rate of butanol and ABE was 92.4 and 93.8%, respectively. The loss of butanol and ABE was mainly due to sample taking and solvents detaining in the recovery system. For PV process, it was clear that the butanol and ABE separation factors and concentrations in condensate were much lower than those in VSVP process. To be highlighted, less time (46 vs. 64 h) was required for VSVP process to make butanol and ABE concentrations in fermentation broth lower than 1 g/L, which contributed to the higher separation factor of VSVP process. Therefore, the VSVP process for butanol recovery was more effective than PV process. In addition, compared with our previous study using corn stover, the demonstrating VSVP process could produce more concentrated butanol (323.4–348.7 g/L vs. 212.0–232.0 g/L) using JAS and optimized conditions [23].

The process simulation of hybrid vapor stripping–vapor permeation (membrane-assisted vapor stripping system, MAVS) indicated that significant reductions in energy demand are possible for MAVS systems compared with conventional distillation systems to separate ABE solvents from butanol/water binary solutions and ABE/water solutions [24]. Furthermore, the MAVS pilot unit shows an excellent demonstration that the energy usage of 10.4 MJ-fuel/kg-butanol is required to achieve 85% butanol recovery from a 1.3% (w/v) solution [43]. Therefore, the VSVP process coupling with ABE fermentation has potential application in industrial production of biobutanol for long duration.

## Conclusions

Biobutanol production by NaOH–H_2_O_2_ pretreated from JAS and its recovery were investigated in this study. The NaOH–H_2_O_2_ pretreatment combined with washing/HCl-neutralization strategy was proved to be effective for improving enzymatic efficacy, butanol yields, as well as reducing wastewater generation by >64%. 11.8 g/L butanol (17.6 g/L ABE) was produced in the best scenario conditions, with increasing butanol (ABE) production by 31.1% (19.7%). The VSVP process was more productive than conventional PV process, which produced 323.4–348.7 g/L butanol (542.7–594.0 g/L ABE) in condensate for in situ product recovery of ABE fermentation. In conclusion, the present study provided important support and strategy for efficient biobutanol production using lignocellulosic biomass.

## Methods

### Pretreatment of JAS

Raw JAS provided by Dalian Tianma Group Co. Ltd. (Dalian, China) was air dried, and then sieved with 24 mesh screen. The chopped dry stalk (10%, w/v) was soaked in different test concentrations of NaOH–H_2_O_2_ solution, and then heated in an autoclave at 121 °C for 60 min. The solid residues were washed with water to remove residual NaOH–H_2_O_2_ in the biomass, and then dried at 60 °C for 24 h. Different amount of water for washing was investigated herein for reducing wastewater generation and improving butanol production. Enzymatic hydrolysis of pretreated JAS was performed in a 250 mL serum bottles with a 100 mL working volume using cellulase (Youtell Biotechnology Co. Ltd, Hunan, China). The solid residues (20%, w/v) were soaked in citric acid buffer (pH4.8) at 50 °C and 150 rpm for 72 h. Finally, the JAS hydrolyzed solution was centrifuged at 6000×*g* for 5 min to remove the precipitate, and then ammonia was used to adjust pH to 6.2, and then stored at 4 °C, until used in subsequent fermentation. Compositional analyses of JAS and NaOH–H_2_O_2_-pretreated JAS were performed following National Renewable Energy Laboratory (NREL) protocol [44, 45].

#### Culture and media

*Clostridium beijerinckii* CC101, an adaptive mutant strain of *C. beijerinckii* NCIMB 8052 (ATCC 51743) obtained by adaption in a fibrous bed bioreactor, was used for ABE fermentation [46]. The seed culture was prepared according to the procedures described previously [46]. The actively growing *C*. *beijerinckii* CC101 cells were incubated at 5% (v/v) and 37 °C with no agitation. The culture bottles, tips, and tubes, etc., were purchased from Dalian Meilun Biotech Co. Ltd. (Dalian, China).

#### ABE fermentation

ABE fermentation was carried out with the P_2_ medium containing a carbon source (JAS hydrolysate) in serum bottles, and other components were described previously [47]. The serum bottles each containing 80 mL medium were sterilized by autoclaving at 121 °C and 15 psig for 15 min. All solutions were purged with nitrogen for 10 min through a sterile 0.2 μm filter, either before or after autoclaving.

#### Preparation of the PDMS membrane

The base solution from the Sylgard^®^184 silicone elastomer kit (Dow Corning, USA) was mixed with the curing agent in the ratio of 10:1 using pentane as the solvent to dilute the mixture. The mixture was stirred completely for 5 min and then 8000×*g* centrifuged for 5 min to wipe off air bubble. The mixture was placed on a cleaning polyvinylidene fluoride (PVDF) plate and cast evenly using a micron film applicator (Paul N. Gardner Company, USA). The mixture on the PVDF plate was then heated in a vacuum oven at 80 °C for 3 h. After the membrane cure, the membrane was carefully peeled off for the VSVP and PV processes. The thickness and area of the PDMS membrane were 200 μm and 58 cm^2^, respectively.

#### The VSVP and PV processes for product recovery

**Fig. 5:**
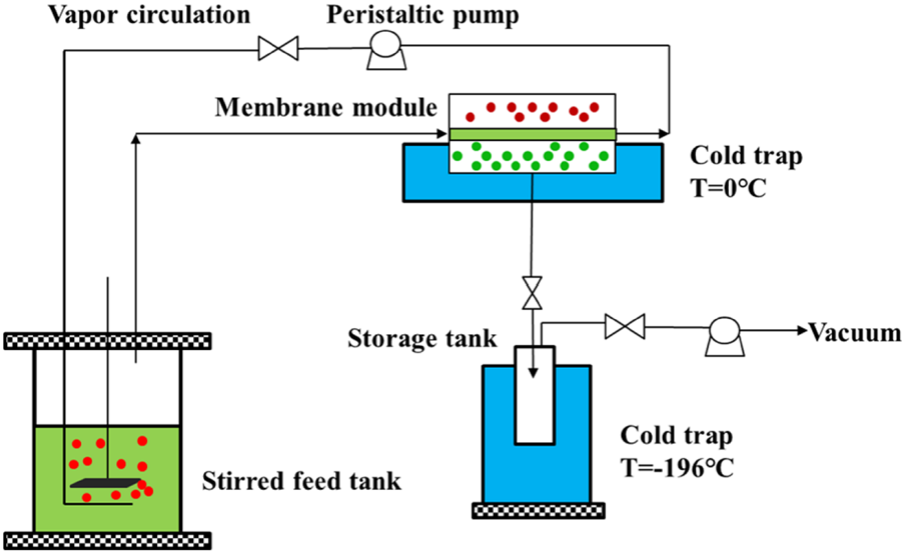
Schematic diagram of the vapor stripping–vapor permeation process apparatus

To mimic in situ product recovery during ABE fermentation, the VSVP and PV processes were carried out using ABE fermentation broth with 500 mL at 37 °C, respectively. The fermentation broth contained 11.8 g/L butanol, 5.7 g/L acetone, and 0.2 g/L ethanol. The vapor stripping–vapor permeation system with a membrane area of 58 cm^2^ is illustrated in Fig. 5. The VSVP and PV processes were compared to recover ABE solvents from the fermentation broth above. The stripping rate for the VSVP process was 2.8 L/min, and the feed fermentation broth for PV was circulated at a flow rate of 2.0 L/min to minimize the boundary layer thickness and maximize mass transfer. The membrane cell was placed in cold bath with ~0 °C. Vacuum was provided on the downstream side of the membrane using a vacuum pump with <100 Pa. The permeate was collected in the storage tank immersed in liquid nitrogen. The flux (ABE and total) and separation factor (SF) were calculated as follows:

$$ {\text{Flux}} = \frac{W}{At}, $$

$$ {\text{SF}} = \frac{{y/\left( {1 - y} \right)}}{x/(1 - x)}, $$

where *W* is the weight of the recovered permeate in gram, *A* is the membrane area in m^2^, and *t* is the time (h) for the sample collection. *x* and *y* are the weight fractions of components in the feed and permeate samples in the VSVP and PV processes, respectively.

#### Analytical methods

The cell density (OD_620_), glucose, butanol, acetone, ethanol, acetic acid, and butyric acid were determined according to our previous study [48]. Various sugars in JAS hydrolysate were analyzed using the HPLC system (Waters 1525) equipped with the column (Aminex HPX-87H, 300 mm × 7.8 mm) operated at 50 °C, photodiode array detector operated at room temperature and 210 nm, and 0.01 mol/L H_2_SO_4_ as the mobile phase with a flow rate of 0.50 mL/min [23].

## Abbreviations

JA: Jerusalem artichoke
JAS: Jerusalem artichoke stalk
ABE: acetone–butanol–ethanol
VSVP: vapor stripping–vapor permeation
MAVS: membrane-assisted vapor stripping system
PV: pervaporation
PVDF: polyvinylidene fluoride
